# Large Roads Reduce Bat Activity across Multiple Species

**DOI:** 10.1371/journal.pone.0096341

**Published:** 2014-05-13

**Authors:** Justin Kitzes, Adina Merenlender

**Affiliations:** Department of Environmental Science, Policy, and Management, University of California, Berkeley, California, United States of America; University of Regina, Canada

## Abstract

Although the negative impacts of roads on many terrestrial vertebrate and bird populations are well documented, there have been few studies of the road ecology of bats. To examine the effects of large roads on bat populations, we used acoustic recorders to survey bat activity along ten 300 m transects bordering three large highways in northern California, applying a newly developed statistical classifier to identify recorded calls to the species level. Nightly counts of bat passes were analyzed with generalized linear mixed models to determine the relationship between bat activity and distance from a road. Total bat activity recorded at points adjacent to roads was found to be approximately one-half the level observed at 300 m. Statistically significant road effects were also found for the Brazilian free-tailed bat (*Tadarida brasiliensis*), big brown bat (*Eptesicus fuscus*), hoary bat (*Lasiurus cinereus*), and silver-haired bat (*Lasionycteris noctivagans*). The road effect was found to be temperature dependent, with hot days both increasing total activity at night and reducing the difference between activity levels near and far from roads. These results suggest that the environmental impacts of road construction may include degradation of bat habitat and that mitigation activities for this habitat loss may be necessary to protect bat populations.

## Introduction

Roads represent one of the most extensive human impacts on the biosphere and are one of the leading causes of habitat fragmentation [1], [2]. Research in the field of road ecology has quantified impacts such as direct mortality due to vehicle collisions, the prevention of physical movement and gene flow across landscapes, and decreases in the use of otherwise suitable habitat [3], [4]. Two recent comprehensive reviews of the road effects literature have found that most studied species showed decreased abundance or activity levels in the vicinity of large roads, with effects extending to several hundred meters from the roadside and beyond [5], [6].

The vast majority of road ecology studies, however, have examined the effects of roads on terrestrial wildlife and birds, and there have been relatively few studies of the effects of roads on bat populations. Several species of bats have been identified in roadkill surveys [7], [8], suggesting that vehicle collisions may be a source of bat mortality. The presence of vehicles on a road has been found to serve as a barrier during commuting and foraging [9], [10], [11], with species-specific responses to passageways across roads having been observed [12]. Traffic noise has been found to reduce the foraging effectiveness of the greater mouse-eared bat (*Myotis myotis*) [13], [14]. Lower levels of total bat activity and activity of the common Pipistrelle (*Pipistrellus pipistrellus*) have also been observed within 1.6 km of a large road in Cumbria, United Kingdom [15].

Here, we present an analysis of the effects of three large roads on bat activity in northern California salt marshes. We combine autonomous recording equipment with a newly developed random forest classifier to investigate the responses of individual bat species to the presence of large roads. The results reveal decreased total bat activity and activity of four common species near roads, suggesting that roads can degrade habitat for North American bats.

## Materials and Methods

### Survey Locations and Methods

Surveys were conducted at a total of ten 300 m transects established at three sites bordering the San Francisco Bay and adjacent to a major highway: Don Edwards San Francisco Bay National Wildlife Refuge (California State Route 84), Hayward Shoreline Interpretive Center (California State Route 92), and San Pablo Bay National Wildlife Refuge (California State Route 37). Average daily traffic at the three roads was 55,000 (SR 84), 86,000 (SR 92), and 33,500 (SR37) vehicles in 2010 [16]. The widths of these roads at locations adjacent to transects are 6–7 lanes and 25–45 m (SR 84), 7 lanes and 40 m (SR 92), and 2 lanes and 15 m (SR 37). All three roads are lit, and the portions of the roads adjacent to transects were level with surrounding habitat (i.e., without bridges or underpasses).

All three study sites are publicly owned and are open for public visitation. Permits for study at the two national wildlife refuges were obtained from refuge headquarters and were granted by the U.S. Fish & Wildlife Service, and permission to sample at Hayward Shoreline was granted by the Hayward Area Recreation and Park District. The passive sampling design of this study did not require contact with individual animals, and as such no permits from the Animal Care and Use Committee at the University of California, Berkeley were required.

Acoustic bat detectors were placed at points located approximately 0–20 m, 100 m, and 300 m from the edge of one side of a road along transects running perpendicular to each highway. Four transects were established at Don Edwards, four at San Pablo Bay, and two at Hayward Shoreline, a smaller site. Habitat was characterized at each transect, with two transects consisting of bare sand and open water (Don Edwards), two of grassy invasive marshland with intermittent trees (San Pablo Bay), four of pickleweed (*Salicornia spp.*) marsh with intermittent trees and shrubs (two at Don Edwards, two at San Pablo Bay), and two of pickleweed marsh (Hayward Shoreline). Within each transect, the habitat type of all sampling points was identical.

Sampling was conducted for a total of 34 nights in August and September of 2010 and 2011, with 5–6 points sampled on each night. Data were collected from sunset to sunrise each night at each sampling point. Although the length of nights varied by as much as one half hour during the course of the study, no clear trends in the number of recorded bat passes with calendar date were observed for either year. Due to logistical constraints and equipment failures, the final sampling design was unbalanced, with each combination of transect and distance sampled for a total of 3–10 nights (Table S1). A total of 174 detector-nights of data were collected.

Surveys were conducted using AnaBat SD2 zero-crossing bat detectors (Titley Scientific Pty Ltd, Lawton, Queensland, Australia) in 2010 and Songmeter SM2BAT 192 kHz full-spectrum bat detectors (Wildlife Acoustics Inc., Concord, MA) in 2011. These detector models are known to have different sensitivities [17], an effect that is controlled for in our statistical models by the year variable (see Statistical Analysis below). Microphones were mounted on 1 m poles to avoid ground noise and reduce recordings of reflected calls. Directional microphones were used with AnaBat detectors and were pointed approximately perpendicular to the transect line and at a 45 degree upward angle. Omnidirectional microphones were used with SM2BAT detectors, and a 6″×6″ foam block was attached to each pole below these microphones to reduce recordings of ground-level insects and noise. The AnaBat detectors were set to autotrigger, saving separate data files for each bat pass, while the SM2BAT detectors recorded data continuously.

### Data Processing and Species Identification

In order to use comparable methods for data analysis across both years, full-spectrum recordings made in 2011 were converted to 8-division zero-crossing format using wac2wav 3.3.0 [18]. A minimum spacing of five seconds was used to separate passes, consistent with the default five second setting used by the AnaBat detectors to separate passes. A single pass was thus defined as a temporally contiguous set of calls with no more than a five second gap between successive calls.

Call data were processed using AnaLookW 3.8s [19]. A filter was used to identify high quality calls, defined as data sequences with a characteristic frequency between 5 and 60 kHz, a duration between 2 and 50 milliseconds, a body over 1 millisecond, and a *Qual* index less than 0.3 [20], [21]. Passes containing no high quality calls were excluded from subsequent analysis. Counts of the number of passes recorded per night were used as a measure of bat activity. For each high quality call, eleven features describing the shape of the call were extracted using AnaLookW [21].

To allow statistical analysis of individual species activity, a random forest classifier was constructed to classify each pass according to the species that produced it, following the methods described in [21]. To begin, a reference library was assembled for 15 bat species known to occur in the San Fransisco Bay area [22] (Table 1). The reference library consisted of AnaBat recordings of bat passes made by individuals of known species, identified in hand (W. Rainey, personal communication). The reference calls and associated features was used to train a random forest classifier, a machine learning algorithm that fits a set of classification trees each built from a bootstrapped sample from a training data set [23], [24]. Among machine learning algorithms, random forests are considered to be highly robust to correlation between predictor variables and do not require specific distributional assumptions about the variation in call features among or between species [23]. All classification analysis was carried out in Python 2.7.2 using scikit-learn 0.11 [25].

**Table 1:** Species present near San Francisco Bay and included in species classifier.

Species	Common Name	N_obs_	N_ref_	*P*	*R*
*Tadarida brasiliensis*	Brazilian free-tailed bat	3372	304	0.86	0.72
*Eptesicus fuscus*	Big brown bat	2094	884	0.83	0.89
*Lasionycteris noctivagans*	Silver-haired bat	1257	365	0.82	0.78
*Lasiurus cinereus*	Hoary bat	582	174	0.91	0.79
*Myotis yumanensis*	Yuma myotis	441	650	0.87	0.96
*Myotis lucifugus*	Little brown bat	196	219	0.75	0.84
*Lasiurus blossevillii*	Western red bat	154	98	0.86	0.64
*Eumops perotis*	Western mastiff bat	51	204	0.98	0.99
*Antrozous pallidus*	Pallid bat	50	353	0.78	0.78
*Parastrellus hesperus*	Canyon bat	45	267	0.96	0.98
*Myotis volans*	Long-legged myotis	26	198	0.79	0.62
*Myotis californicus*	California myotis	23	220	0.75	0.54
*Corynorhinus townsendii*	Townsend's big-eared bat	6	148	0.86	0.77
*Myotis evotis*	Long-eared myotis	2	169	0.83	0.83
*Myotis thysanodes*	Fringed myotis	0	184	0.89	0.91
Total		8299	4437		

N_obs_ is the number of passes recorded during the study that were identified as each species. Counts for less abundance species should be interpreted with caution due to uncertainties in the classification method. Remaining columns evaluate the effectiveness of the statistical classifier used to identify species from call data. N_ref_ is the number of passes for each species included in the reference library. *P* is precision, the fraction of all calls in the testing data set identified as a species that were made by that species, and *R* is recall, the fraction of all calls in the testing data set made by a species that were identified as that species. Overall species classification accuracy, the fraction of all calls in the testing data set identified correctly, was 84%.

A classifier consisting of 100 trees was trained on 80% of reference calls, selected using stratified random sampling to achieve proportional representation of each species, with 20% of calls held out for testing. All trees were fully built, with three randomly drawn features used to split each node. The use of larger ensembles of trees did not substantially improve classification accuracy. Ten replicate fits of the classifier had a mean accuracy (proportion of calls in the testing set correctly identified) of 0.8411 (SD 0.0013) (Table 1), similar to the accuracy achieved by random forest classifiers constructed for southwestern and Florida bats [21].

The fitted random forest classifier was used to predict the probability that each high quality call recorded during the field surveys was made by each species. These probabilities were summed across all calls within each pass, and the species with the highest summed probability for a pass was identified as the species associated with that pass (Table S2). Summed counts of the total number of passes per night were used for all subsequent species-level analysis.

Classifying recorded bat passes to the species level is a challenging task due to the presence of call fragments, overlapping calls made by multiple individuals, within–species variability in call structure, and other factors [26]. While much effort has been devoted to developing methods that account for these complexities [27], analyses based on species–level acoustic identifications must proceed carefully in light of the limitations of call classification methods. Given the relatively accurate yet imperfect performance of our fitted classifier, several decisions of study design were made to improve the reliability of our overall findings. First, a random forest classifier was selected over other common approaches, including discriminant function analysis, artificial neural networks, and supported vector machines, as the random forest approach was recently recommended in a comparative analysis of bat call classifiers [21]. Second, no species-level analysis was conducted for species in the genus *Myotis*, as the species of this genus present in California are known to be particularly difficult to distinguish based on echolocation calls. Third, species–level analysis was not attempted for any species with fewer than 500 identified passes, and presence–absence and species richness analyses were avoided.

In addition to the above, the importance of classifier accuracy was also tested quantitatively by adjusting the criteria used to include a pass in a species–level model. A logical probability threshold for including a pass in a species model is a mean call probability of 50%, indicating that the classifier assigned a greater probability to the chosen species than to all other species combined. As our statistical models did not converge for all species when a 50% threshold was applied to the pass data, a 45% threshold was tested for all four individual species analyzed in this study. Approximately two-thirds of the passes recorded in our survey were assigned to a species at this 45% threshold, and the results of our statistical models for this reduced dataset were comparable to the results presented in Table 2. In particular, the magnitude of the estimated road effect for each species was similar in both analyses and significant at the *P*<0.01 level.

**Table 2:** Results of negative binomial generalized linear mixed models of counts of nightly bat passes.

Coefficient	All species		T. brasilensis		E. fuscus		L. noctivagans		L. cinereus	
Intercept	3.4870	**	2.6711	**	0.2755	**	−0.0029	**	1.0646	**
dist_road		**		**		**		**		**
dist_road_100	−0.3560		−0.2624		−0.4410		−0.5513		−0.3051	
dist_road_0	−0.6853		−0.6459		−0.5909		−1.0851		−0.5279	
temp_max	0.0529	*	0.0428	.	0.0874	**	0.0510	*	0.0482	.
dist_road:temp_max		*		.		.		.		.
dist_road_100:temp_max	−0.0429		−0.0460		0.0735		0.0119		−0.0509	
dist_road_0:temp_max	0.0499		0.0316		0.1177		0.1558		0.0289	
site		**		**		**		**		*
site1	−0.3937		−0.4017		−0.0068		−1.1843		−0.1025	
site2	−1.0882		−0.8379		−3.5913		−2.0287		0.1845	
dist_road:site		**		**		**		*		*
dist_road_100:site1	0.5899		0.8801		0.1765		0.5549		0.6708	
dist_road_0:site1	0.3101		0.4048		0.0225		0.2496		0.0976	
dist_road_100:site2	−0.3017		−0.4784		0.3632		−0.1876		−0.6392	
dist_road_0:site2	1.0328		0.5164		2.1542		1.3285		0.4569	
year	0.1384	.	0.4238	*	−0.8726	**	−0.6150	*	0.4750	**
light	0.2129	.	0.0849	.	0.2592	.	0.4402	.	0.2159	.

Variables are distance from the road, maximum daily temperature, site, year, and presence of a light within 100 m (fixed effects), and night and transect (random effects, not shown). * indicates p-values below 0.05 and ** indicates p-values below 0.01 as determined by a likelihood ratio test. Rows without a symbol represent levels of categorical variables whose significance could not be tested individually. Distance coefficients are relative to a control distance of 300 m. Coefficients are in units of ln(passes).

Finally, we note that our analysis of total bat activity is unaffected by the uncertainties inherent in species classification, as the classifier is used here only to divide passes into species groups for the species-level models.

### Statistical Analysis

Statistical analyses were carried out using generalized linear mixed models (GLMMs) [28], [29] using R 2.14.2 [30] with the packages lme4 0.999375-42 [31] for Poisson models and glmmADMB 0.7.2.12 [32], [33] for negative binomial models.

The statistical models included three main variables of interest that could potentially influence total bat activity: distance from road (*dist_road*, three levels: 0 m, 100 m, and 300 m), presence of a light within 100 m of a sampling point (*light_100*, two levels: False, True), and daily maximum temperature (*temp_max*, continuous). The latter two variables were included due to substantial prior research that shows that light sources [34], [35], [36], [37] and ambient temperature [38], [39], [40] can affect bat activity levels. Lights were present within 100 m of four out of thirty sampled points.

Daily maximum temperature data were drawn from records at the KOAK weather station at the Oakland International Airport, located on the shore of San Francisco Bay [41]. As ambient temperature at locations around the margins of San Francisco Bay are very similar, and all three sites were immediately adjacent to the bay, temperature data recorded at this station were used as an estimate of daily maximum temperature at all sampled locations. Although wind speed data were also available from this station, mean daily wind speed and daily maximum temperature were relatively strongly correlated during the nights of our study (univariate log-log regression *β* = −1.90, R^2^ = 0.52), and, as such, a wind speed variable was not included in regression models.

In addition to these three main variables, four additional variables representing site (*site*, three levels: DOED, HAYW, SAPA), year (*year*, two levels: 2010, 2011), night (*night*, random effect), and transect (*transect*, random effect) were also included in statistical models. The site and year variables were coded as fixed effects, as only three and two levels of these variables were present, respectively. Because sampling was carried out using different detector models in 2010 and 2011, the estimated effect of year also includes the effect of detector model. Additional interaction terms between *dist_road* and *site* and between *dist_road* and *temp_max* were added to account for the biologically-reasonable possibility that road effects may vary by site and with temperature. With all variables and interactions, the final models had a total of 14 estimated fixed effect parameters (Table 1) plus normally distributed crossed random effects for night and transect.

As appropriate for the analysis of count data, exploratory analyses were conducted using Poisson generalized linear mixed models [29], [42]. Initial models containing all predictor variables described above indicated that nightly count data were overdispersed, with the ratio of summed squared Pearson residuals to residual degrees of freedom (accounting for fixed effects only) equal to 13.67 for a model including all passes and ranging from 2.57 to 6.06 for individual species models (see *Results*). To account for this overdispersion, negative binomial generalized linear mixed models, specified with type 1 variance structure within the glmmADMB package and using a log-link function, were used for subsequent modeling.

In specification of the model matrix, sum contrasts were used for the variables *road*, *year*, and *light_100*, such that the sum of estimated coefficients for all levels of each variable are equal to zero, as there is, philosophically, no control level of these variables. Treatment contrasts were used for *dist_road*, with counts at 300 m from the road serving as the control level. Temperature data were centered on the mean maximum daily temperature (24°C).

Given this model matrix, the estimated intercept gives the number of bat passes expected at a distance of 300 m at the mean daily maximum temperature. Estimates of the number of passes at 0 m and 100 m are obtained by adding the *dist_road_0* and *dist_road_100* coefficients, respectively. The addition of the coefficient *site1* gives estimates for San Pablo Bay, the addition of the coefficient *site2* gives estimates for Don Edwards, and the subtraction of both coefficients gives estimates for Hayward Shoreline. Estimates of passes in 2010 are obtained by adding the coefficient *year*, and estimates of passes in 2011 are obtained by subtracting this coefficient. Estimates of passes at locations within 100 m of a light are obtained by subtracting the coefficient *light*, and estimates of passes at locations more than 100 m from a light are obtained by adding this coefficient. Similar logic applies to the interpretation of the coefficients of the interaction terms.

The significance of each predictor variable was tested using likelihood ratio tests of the full model containing all predictors and a reduced model omitting only that predictor, carried out using the *anova* command of the package glmmADMB. Reduced models used to test the direct effects of road, distance from road, and temperature necessarily excluded the interaction terms that included those variables. Reduced models used to test interaction terms retained all direct effects.

## Results

A total of 8,299 bat passes were recorded across 174 detector-nights of sampling. Of all recorded passes, more than 85% were identified by the random forest classifier as either *Tadarida brasiliensis*, the Brazilian free-tailed bat (40%), *Eptesicus fuscus*, the big brown bat (25%), *Lasionycteris noctivagans*, the silver-haired bat (15%), or *Lasiurus cinereus*, the hoary bat (7%) (Table 1).

**Figure 1:**
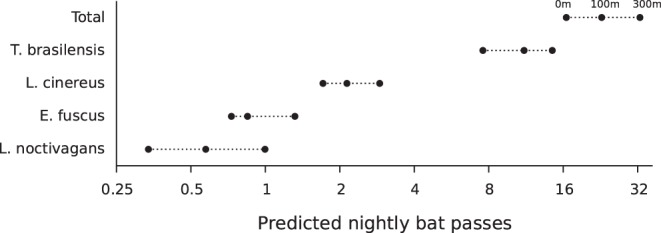
Model predictions of nightly bat passes by species and distance from road. Three values are displayed for each species, representing the predicted activity for that species at 0 m, 100 m and 300 m from the road. All models show an approximate doubling of bat activity at 300 m as compared to 0 m with the exception of *L. noctivagans*, which shows a tripling.

Statistical models of bat activity, measured as a nightly count of bat passes, were fit for the total number of bat passes made by all species and separately for each of the four most common species in this landscape (Table 2). Across all transects and nights, and for all species, predicted activity was lowest at 0 m from the road, highest at 300 m from the road, and intermediate at 100 m (Fig. 1). Total predicted bat activity was approximately twice as high at 300 m from the road than at 0 m, with predictions of 16.5 passes per night at 0 m, 22.9 passes per night at 100 m, and 32.7 passes per night at 300 m from the road edge. A road effect of similar magnitude is predicted for *T. brasiliensis*, *E. fuscus*, and *L. cinereus*, while a larger effect (approximately a tripling of activity at 300 m compared to 0 m) is predicted for *L. noctivagans*. A likelihood ratio test shows that the effect of distance from a road is statistically significant for all species.

Maximum daily temperature prior to the recording night varied greatly during the course of the surveys, ranging from 17°C to 37°C with a mean of 23°C (SD±4.9°C). Temperature was found to significantly influence total bat activity and activity of *E. fuscus* and *L. noctivagans*, with increasing temperature associated with an increase in recorded bat passes. A significant interaction between distance from a road and temperature was also observed for the model of total activity, with the difference between activity levels at 0 m and at 300 m decreasing with an increase in temperature. This same qualitative pattern was observed for the four individual species, although the distance-temperature interaction was not statistically significant in any of the species-level models.

The number of recorded bat passes varied greatly by site for all species. Transects at Hayward Shoreline had substantially higher total bat activity than those at Don Edwards or San Pablo Bay as well as the strongest road effects. The interaction of distance and site was also significant for all models, suggesting that the road effect varied by site. The effect of year was not significant for the model of total activity but was significant for each individual species, with more *T. brasiliensis* and *L. cinereus* passes recorded in 2010 with the AnaBat detectors and more *E. fuscus* and *L. noctivagans* passes recorded in 2011 with the SM2BAT detectors. These differences may reflect either year-to-year variability in activity levels or different sensitivities of these two detector models to the calls of different species. The presence of a light within 100 m of a sampling point did not have a statistically significant effect on total bat activity or the activity of any individual species.

## Discussion

These results demonstrate that total bat activity and the activity of four common bat species is consistently depressed near three large California highways as compared to control points 300 m from these roads. Possible explanations for this road effect include differences in traffic noise, headlight illumination, wind speed, temperature, or prey availability between points near and far from roads, as well as the overall barrier effect of the road itself [15]. While this study was designed only to test for the existence of a road effect and not to quantitatively examine its potential causes, distinguishing between these causes will be an important future area of research.

Although many previous studies have documented increases in bat activity with increasing ambient temperature [38], [39], [40], a significant interaction between temperature and road avoidance in bats has not been previously described to our knowledge. On the hottest days of the study (daily maximum 37°C), models for all species predict approximately constant activity across all distances from a road, with road effects increasing in magnitude as temperature decreases. This pattern could potentially result from crowding if higher overall bat densities lead bats to spread out across the landscape, increasing activity in otherwise less desirable habitat, or if road surfaces themselves retained more heat following hot days, perhaps increasing insect activity near the roads during the subsequent night. Sound attenuation is also generally expected to be higher in higher-temperature environments, and reduced traffic noise near roads may also play a role in the increased activity levels observed near roads at higher temperatures. More generally, the statistically and biologically significant effect of temperature on total bat activity, and the significant interactions between temperature and the road effect, suggests that future road ecology studies for bats should explicitly test for this interaction between road avoidance and temperature.

Our results corroborate those of Berthinussen and Altringham [15], who found a significant effect of distance from a road on total bat activity and the activity of *Pipistrellus pipistrellus* near a large road in the United Kingdom. The road effect found in our study, however, operated over a much shorter distance, with the approximately two-fold difference in activity between 0 m and 300 m in our study being comparable to an effect of the same magnitude between points at 0 m and 1000 m from the roadside in this previous study. The Berthinussen and Altringham [15] study did not report investigations of the potential for an interaction between temperature and the road effect, which is an important result of our analysis.

Several limitations of this study are important to note and suggest directions for future research. First, the scope of this study was limited to three roads and four individual species. Larger scale studies that evaluate different habitat types and species, including those that are rarer and potentially more disturbance-sensitive, would be needed to generalize these findings more broadly. Second, as described above, the results presented here are correlative, as this study was not designed to explore or test the mechanisms behind the observed road effect. An understanding of these mechanisms will be a necessary precursor to any attempts to change management, planning, or mitigation activities to protect bat habitat adjacent to roads. Third, as bat activity was only sampled to a distance of 300 m from the road side, these distances have been used as controls in comparisons to activity levels at 0 m. The actual road effects may extend farther than 300 m [15], however, leading to an underestimate of the decrease in bat activity near roadsides when compared with core natural areas. Finally, the results for individual species involve uncertainties related to species identification, as no classification method, including the one used here, is able to perfectly identify bat calls to the species level.

North American bat populations face well-known threats from habitat loss [43], the emerging disease white-nose syndrome [44], and wind energy development [45]. Our results suggest that habitat degradation due to roads may be a significant and previously under-appreciated threat to bat populations. These results provide the first quantitative evidence that road construction and improvement projects should consider the possible degradation of North American bat habitat that accompanies these activities.

## Supporting Information

Table S1 **Summarized bat passes by site, transect, road distance, date, and species.**
(CSV)

Table S2 **Recorded bat passes with classification probability.** Row labels indicate site, transect, road distance, date, and time of bat pass recording, and columns indicate summed classification probability for a species across all calls in a pass. Passes were assigned to the species with maximum summed probability. The sum of probabilities across all species for a pass equals the number of calls in that pass.
(CSV)
