# Correction: Large roads reduce bat activity across multiple species

**DOI:** 10.1371/journal.pone.0105388

**Published:** 2014-08-15

**Authors:** The PLOS ONE Staff

Table 2 is formatted incorrectly. Please see the correct Table 2 here.

**Table:** 

Coefficient	All species		T. brasiliensis		E. fuscus		L. noctivagans		L. cinereus	
Intercept	3.4870	**	2.6711	**	0.2755	**	-0.0029	**	1.0646	**
dist_road		**		**		**		**		**
dist_road_100	-0.3560		-0.2624		-0.4410		-0.5513		-0.3051	
dist_road_0	-0.6853		-0.6459		-0.5909		-1.0851		-0.5279	
temp_max	0.0529	*	0.0428	.	0.0874	**	0.0510	*	0.0482	.
dist_road:temp_max		*		.		.		.		.
dist_road_100:temp_max	-0.0429		-0.0460		0.0735		0.0119		-0.0509	
dist_road_0:temp_max	0.0499		0.0316		0.1177		0.1558		0.0289	
site		**		**		**		**		*
site1	-0.3937		-0.4017		-0.0068		-1.1843		-0.1025	
site2	-1.0882		-0.8379		-3.5913		-2.0287		0.1845	
dist_road:site		**		**		**		*		*
dist_road_100:site1	0.5899		0.8801		0.1765		0.5549		0.6708	
dist_road_0:site1	0.3101		0.4048		0.0225		0.2496		0.0976	
dist_road_100:site2	-0.3017		-0.4784		0.3632		-0.1876		-0.6392	
dist_road_0:site2	1.0328		0.5164		2.1542		1.3285		0.4569	
year	0.1384	.	0.4238	*	-0.8726	**	-0.6150	*	0.4750	**
light	0.2129	.	0.0849	.	0.2592	.	0.4402	.	0.2159	.
